# Clinical Features and Outcomes in Pediatric Autoimmune Encephalitis Associated With CASPR2 Antibody

**DOI:** 10.3389/fped.2021.736035

**Published:** 2021-10-01

**Authors:** Chengbing Tan, Yan Jiang, Min Zhong, Yue Hu, Siqi Hong, Xiujuan Li, Li Jiang

**Affiliations:** ^1^Department of Neurology, Children's Hospital of Chongqing Medical University, National Clinical Research Center for Child Health and Disorders, Ministry of Education Key Laboratory of Child Development and Disorders, Chongqing, China; ^2^Chongqing Key Laboratory of Translational Medical Research in Cognitive Development and Learning and Memory Disorders, Chongqing, China

**Keywords:** autoimmune encephalitis, contactin-associated protein-like 2, clinical features, outcomes, children

## Abstract

**Background:** Contactin-associated protein-like 2 (CASPR2) neurological autoimmunity has been associated with various clinical syndromes involving central and peripheral nervous system. CASPR2 antibody-associated autoimmune encephalitis is mostly reported in adults. Analysis of the clinical presentation and prognostic data of CASPR2 antibody-associated autoimmune encephalitis in children remains important.

**Methods:** A single-center retrospective review of children diagnosed with CASPR2 antibody-associated autoimmune encephalitis from June 1st, 2018 to October 31st, 2020.

**Results:** Six patients were identified. The median age was 12 years (range 1.8–14), with an overall male predominance of 83% (5/6). Commonest clinical features were psychiatric symptoms (6/6), movement disorders (4/6), altered consciousness (3/6), sleep disorders (3/6), and headache (3/6). Four patients (4/6) received first-line therapy alone (steroids combined with intravenous immunoglobulins), and two patients (2/6) received second-line therapy (rituximab, mycophenolate mofetil, or cyclophosphamide). All patients showed no peripheral nervous system involvement. One patient had comorbidities with systemic lupus erythematosus. No evidence of neoplastic disease was found in the whole cohort. All patients had favorable outcomes (modified Rankin Score 0–2) with recurrence rate at 0%, respectively.

**Conclusion:** CASPR2 antibody-associated autoimmune encephalitis is rare in children. Our findings suggest that this type of encephalitis seems to occur more frequently in older children. Patients respond well to immunotherapy and usually demonstrate a favorable clinical outcome. Associated tumors are extremely rare.

## Introduction

Autoimmune encephalitis (AE) refers to a group of antibody-mediated inflammatory diseases of the brain. The major neurologic manifestations of AE include altered level of consciousness, confusion, sleep disturbances, movement disorders, seizures or autonomic dysfunction. To date, several antibodies against neuronal cell-surface or synaptic proteins have been identified (1). Voltage-gated potassium channels (VGKCs) are present on the membrane of neurons in both the central and peripheral nervous system which were initially detected in rare patients suffering from neuromyotonia (NMT) in 1995. Subsequently, VGKC antibodies were found in patients with Morvan syndrome (MoS) which mixes NMT and encephalopathy, and in patients with limbic encephalitis. Leucine-rich glioma-inactivated protein 1 (LGI1) and contactin-associated protein-like 2 (CASPR2) were identified as the main antigens within the VGKC in 2010 (2). CASPR2 is a membrane protein that is widely expressed in neurological sites such as the cortex, limbic system, basal ganglia and sensory organs. CASPR2-associated diseases mainly include gene-associated neurodevelopmental diseases and antibody-associated autoimmune diseases. Mutations in the gene encoding CASPR2 have been reported in patients with autism spectrum disorder, epilepsy and other neuropsychiatric problems. The clinical spectrum of autoimmune diseases associated with anti-CASPR2 antibody can manifest as encephalitis, NMT, MoS and painful neuropathy, and are mostly reported in adults (3). Pediatric manifestations of CASPR2 autoimmunity are less well studied and limited to single case reports or small case series (4). In the present study, we report 6 pediatric cases of CASPR2 antibody-associated autoimmune encephalitis.

## Methods

We retrospectively collected clinical data from patients with autoimmune encephalitis hospitalized at Children's Hospital of Chongqing Medical University, the largest tertiary pediatric medical center in southwest China, from June 1st, 2018 to October 31st, 2020. All the patients included in the study met the pediatric autoimmune encephalitis criteria as follows (5): (1) Onset of neurologic and/or psychiatric symptoms over ≤3 months in a previously healthy child; (2) Two or more clinical features including altered mental status/level of consciousness or EEG with slowing or epileptiform activity, focal neurologic deficits, cognitive difficulties, acute developmental regression, movement disorder, psychiatric symptoms, seizures not explained by a previously known seizure disorder or other condition; (3) Two or more neuroinflammation features including CSF inflammatory changes (leukocytosis >5 cells/mm^3^ and/or oligoclonal banding), MRI features of encephalitis, brain biopsy showing inflammatory infiltrates and excluding other disorders; (4) Presence in serum and/or CSF of well-characterized autoantibodies associated with AE; (5) Reasonable exclusion of alternative causes. Other etiologies such as infectious, metabolic, toxic and hereditary diseases were excluded based on clinical history and necessary auxiliary examinations.

Detailed clinical examination, imaging examination and cerebrospinal fluid (CSF) examination were used to confirm the diagnosis. Specimens were tested for CASPR2-IgG by a third-party clinical laboratory center. Sera or CSF Caspr2-IgG were detected by a cell-based assay (CBA) using human embryonic kidney (HEK293T) cells transiently co-transfected with full-length human Caspr2 and pcDNA3.1-EGFP. After 36 h of transfection in 96-well plate, the cells were fixed with 4% paraformaldehyde for 20 min, and ready for antibody detection. Sera were diluted at 1:10 in PBS-10% goat serum and incubated on cells for 2 h at room temperature. Cells were then washed in PBS-0.1% Tween 20 for 3 times, incubated for 30 min with goat anti-human IgG (1:500, Thermo Scientific), washed again in PBS-0.1% Tween 20, and evaluated by immunofluorescence microscopy. Two independent masked assessors classified each sample as positive or negative based on the intensity of surface immunofluorescence in direct comparison with non-transfected cells and control samples. Once confirmed, the Caspr2+ sera were then serially diluted by three-fold from 1:10 to 1:1,000 to determine the titers. The final titer was defined as the sample dilution value for which specific fluorescence was barely but clearly identifiable and expressed as the corresponding dilution value. In parallel, specific antibodies including N-methyl-d-aspartate receptor (NMDAR), α-amino-3-hydroxy-5-methyl-4-isoxazolepropionic acid receptor (AMPAR), leucine-rich glioma-inactivated protein 1 (LGI1) and γ-aminobutyric acid type B receptor (GABABR) were evaluated. Autoimmune and tumor-related assessments included autoantibody series, antinuclear antibody profile, serum complement levels, thyroid function, Chest-abdomen computed tomography (CT)-enhanced scan, and tumor markers (human chorionic gonadotropin, carcinoembryonic antigen, neuron specific enolase, and vanillylmandelic acid). Necessary tests were performed to exclude infections, including cerebrospinal fluid culture and staining (bacterial, tuberculosis, and fungal), PCR and antibody testing of serum and cerebrospinal fluid (herpes simplex virus, Epstein-Barr virus, enterovirus, and Mycoplasma pneumoniae), and serological testing for HIV and syphilis. Patients with unexplained psychiatric symptoms were also tested for autoimmune encephalitis-related antibodies.

The modified Rankin Scale (mRS) score was used to retrospectively assess the neurological severity of the acute phase and the outcome of the final follow-up (6). The follow-up was conducted through outpatient visits or telephone interviews. Long-term good prognosis was defined as mRS score ≤ 2, and long-term poor prognosis was defined as mRS score > 2. Relapse was defined as recurrence of symptoms after full or partial recovery, with sustained improvement for at least 2 months (7).

Statistical analyses were performed using SPSS statistical software (IBM, New York, New York, 2019). Descriptive statistics were applied to analyze clinical data, such as medians and percentages.

## Results

### Demographic and Clinical Features

Positive autoimmune encephalitis antibodies were detected in 103 of 674 suspected patients. Nine patients were positive for anti-CASPR2 antibodies. Two patients presented only with acute onset of psychiatric symptoms, which resolved spontaneously within 1 week without any treatment. Another patient presented with drowsiness and acute cerebellar ataxia, which was self-limited within 1 week without immunotherapy. Therefore, the above three patients with a serum antibody titer of 1:10 were excluded from the present study. Six patients with positive anti-CASPR2 antibody were diagnosed with autoimmune encephalitis and included in this study.

Demographic and clinical features are shown in Table 1. The median age was 12 years (range 1.8–14). The male to female ratio was 5:1. The initial symptoms were psychiatric symptoms (6/6), frequent seizures (1/6), fever (2/6), and headache (1/6). The common clinical manifestations were psychiatric symptoms (6/6), movement disorders (4/6), altered consciousness (3/6), sleep disorders (3/6), headache (3/6), fever (2/6), seizures (2/6), and speech disorders (1/6) during the course of the disease. In this group of patients, the main psychiatric symptoms were hallucinations, babbling, aggressive behavior, disruptive behavior, mania, or personality changes. Movement disorders included orofacial stereotypies (1/6) and choreoathetosis involving the upper extremities (3/6). Dysautonomia and peripheral nervous system symptoms were absent. Severe neurological dysfunction was present at the peak of the disease (mRS range 3–4). One patient (patient 1, Table 1) who developed facial butterfly erythema and oral ulcer showed antinuclear antibodies and anti-dsDNA antibody positive in serum. In another patient (patient 5, Table 1), obsolete pulmonary tuberculosis was diagnosed due to obsolete lung lesion on chest enhanced CT scan, positive interferon gamma release assay, and positive tuberculin skin test without evidence of active tuberculosis infection.

**Table 1:** Clinical and investigatory findings of patients with positive anti-CASPR2 antibody.

**No**.	**Age, Y/Sex**	**Clinical features**	**Cranial MRI**	**EEG**	**CSF**	**CASPR2-IgG titer**
1	12.8/F	Fever, headache, altered consciousness, seizures, psychiatric symptoms, facial butterfly erythema, oral ulcer	Normal	Slow background activity (delta range); epileptiform discharge	WBC 4 Prot 41	Serum 1:10
2	11.6/M	Headache, psychiatric symptoms, sleep disorders, movement disorders	Normal	Normal	WBC 11 Prot 28	Serum 1:30
3	12.3/M	Fever, headache, psychiatric symptoms, sleep disorders, hypertension, movement disorders	Normal	Focal slow waves	WBC 14 Prot 102	Serum 1:10
4	1.8/M	Seizures, psychiatric symptoms, movement disorders	Normal	Focal slow waves	WBC 2 Prot 25	Serum 1:10
5	14/M	Altered consciousness, psychiatric symptoms, sleep disorders, aphasia	Abnormal	Slow background activity (theta range)	WBC 0 Prot 35	Serum 1:10
6	6.9/M	Altered consciousness, psychiatric symptoms, movement disorders	Normal	Slow background activity (theta range)	WBC 0 Prot 15	Serum 1:30

*Y, years; F, female; M, male; CSF, cerebrospinal fluid; MRI, magnetic resonance imaging; EEG, electroencephalogram; WBC, white blood cells/mm^3^ in CSF; Prot, CSF total protein (mg/dl); CASPR2-IgG, contactin-associated protein-like 2 IgG*.

*Focal demyelination of the bilateral frontal lobes*.

**Figure 1:**
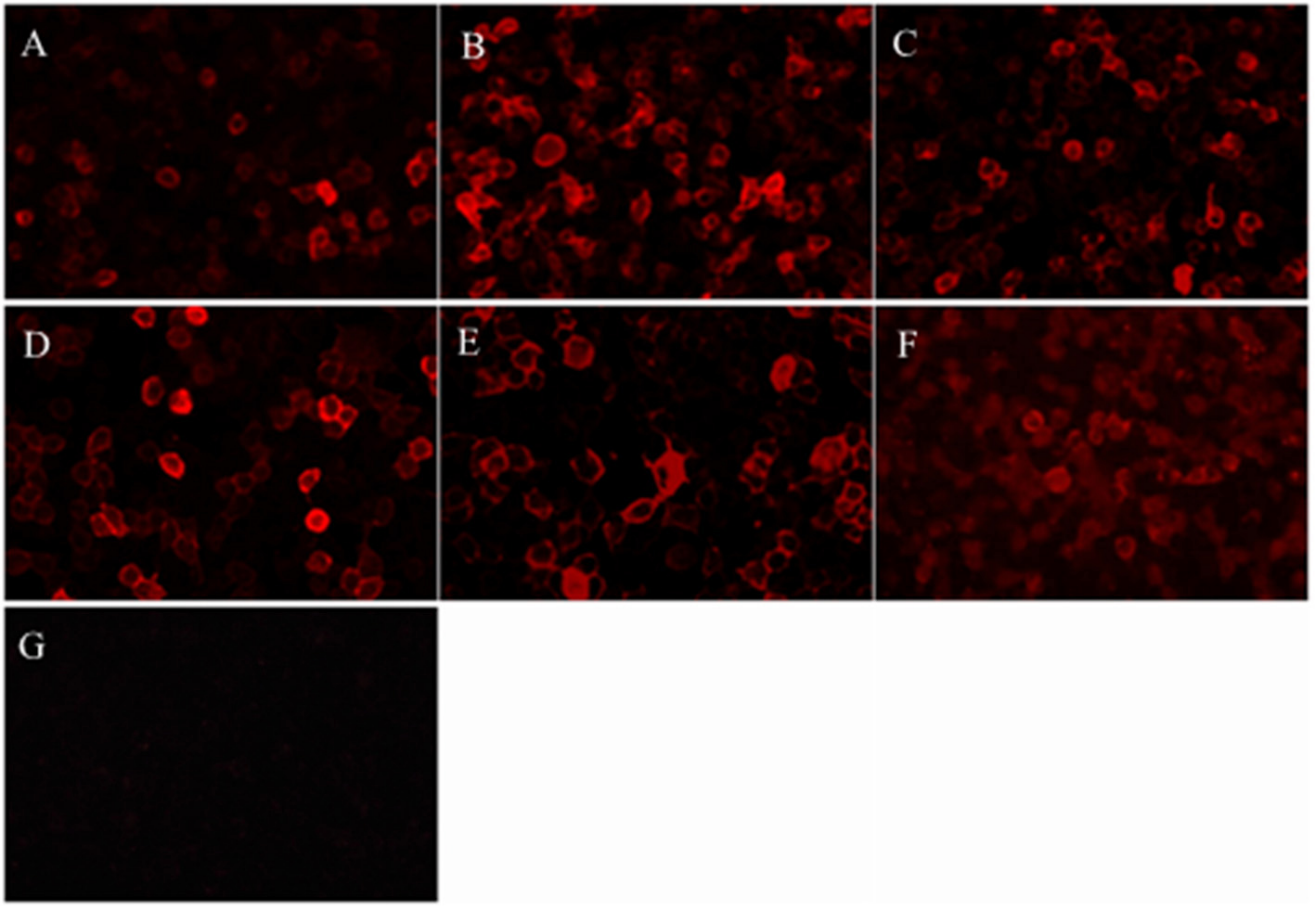
Patients' antibodies reacted with HEK293 cells expressing CASPR2. **(A-F)** Diluted serum samples of the patient 1–6, respectively, reacted with HEK cells transfected with plasmid encoding human CASPR2 gene. **(G)** A sample from the control patient shows the negative labeling.

Focal demyelination lesions of the bilateral frontal lobes in cranial magnetic resonance imaging (MRI) was found in one patient and cranial MRI were normal in other five patients. Chest-abdomen CT-enhanced scan and tumor markers showed no evidence of neoplastic disease. Thyroid function analysis such as anti-thyroglobulin antibody and anti-thyroid peroxidase antibody was normal. Serum and cerebrospinal fluid tests were negative for viruses, bacteria and fungi. CASPR2-IgG were positive in all sera samples but negative in CSF samples (Table 1 and Figure 1). Electroencephalogram demonstrated generalized or focal slow waves in 5 patients.

### Treatment and Follow-Up

The first-line immunotherapy strategy in five patients used high-dose intravenous methylprednisolone (IVMP, 20 mg/kg, maximum dose 1,000 mg once daily for 3 ~ 5 days) combined with intravenous immunoglobulin (IVIG, 1 g/kg once daily for 2 days). Prednisone acetate (1 mg/kg, maximum dose 60 mg daily) were given orally after methylprednisolone treatment. Another patient (patient 2, Table 2) improved significantly after treatment with IVIG and was subsequently given oral prednisone acetate because the guardian refused to use intravenous methylprednisolone. However, 1 month later the patient developed psychiatric symptoms and sleep disturbances again. The repeat examination were negative for anti-CASPR2 antibody and normal for cranial MRI. The condition improved after steroids (IVMP and prednisone acetate) and IVIG therapy. Two patients received second-line immunotherapy with rituximab, mycophenolate mofetil or cyclophosphamide (Table 2). Four patients received other treatment as the following: 2 antiepileptic drugs, 1 anti-tuberculous therapy, and 3 risperidone (Table 2).

**Table 2:** Treatment and follow-up of patients with positive anti-CASPR2 antibody.

**No**.	**Therapy**		**Final follow-up, months**	mRS score at follow-up (vs. acute phase)/Time^*^

	**Immune therapy**	**AEDs and other therapy**		
1	IVIG, IVMP, prednisone, CPA^#^	OXC, NZP, LEV	20	0 (4)/1
2	IVIG, IVMP, prednisone	Risperidone	23	0 (3)/9
3	IVIG, IVMP, prednisone	Risperidone	14	0 (3)/6
4	IVIG, IVMP, prednisone		13	2 (3)/6
5	IVIG, IVMP, prednisone, RTX, MMF	OXC, risperidone, anti-tuberculous therapy^††^	7	0 (3)/5
6	IVIG, IVMP, prednisone		7	0 (3)/3

*IVIG, intravenous immunoglobulins; IVMP, intravenous methylprednisolone; RTX, rituximab; MMF, mycophenolate mofetil; AEDs, antiepileptic drugs; OXC, oxcarbazepine; NZP, Nitrazepam; LEV, levetiracetam; CPA, cyclophosphamide*.

* *Time is defined as the time in months to reach the final mRS score during the follow-up period*.

# *The patient with an overlap of CASPR2-associated autoimmune encephalitis and systemic lupus erythematosus was given cyclophosphamide infusion therapy for systemic lupus erythematosus*.

†† *The patient was given anti-tuberculous therapy for obsolete pulmonary tuberculosis*.

Clinical follow-up data were available from all patients (median 14 months, range 7–23months). The vast majority of patients achieve a stable neurological function within half a year of the disease course (median 5.5 months, range 1–9 months) (Table 2).Five patients showed good neurologic response to immunotherapy with a mRS score 0. One patients had partial response with a mRS score 2. Prednisone acetate therapy were tapered for 4–6 months, except for one patient who required long-term low-dose maintenance for SLE. All patients did not experience recurrence at the time of final follow-up.

## Discussion

The fact that antibodies against VGKC-complex can lead to autoimmune encephalitis was confirmed (7). Hundreds of cases of CASPR2 antibody-associated neurological disorders have been reported in the last decade. A systematic review showed that the most common reported clinical syndromes were autoimmune encephalitis (69/134, 51.5%) and limbic encephalitis (106/274, 38.7%). Peripheral nerve hyperexcitability (72/191, 37.7%) and Morvan syndrome (57/251, 22.7%) were also reported (8). Neurological disorders associated with CASPR2 antibodies occur mainly in adults. Cognitive dysfunction is the most common manifestation in adult patients with CASPR2-associated AE, with <30% experiencing psychosis and 16–55% experiencing peripheral hyperexcitability (2, 7, 9). The proportion of autoimmune encephalitis and peripheral nervous system involvement is lower in children compared to adults. Pediatric patients with a phenotype of pure autoimmune encephalitis are even rarer. A systematic review of LGI1 and CASPR2-related diseases in children showed that 57.1% of CASPR2 positive patients presented with isolated epilepsy, epileptic encephalopathy or seizure disorder, and only 14.3% (2/14) presented with simple encephalitis. Syndromes with mixed central nervous system and peripheral nervous system involvement were 14.3%, with no reports of Morvan syndrome (10). To date, a total of more than 20 cases of CASPR2 autoimmunity in children have been reported, and only a few cases of autoimmune encephalitis have been adequately described (4, 11–14). In the present study, all patients had a clinical syndrome of autoimmune encephalitis with no signs of peripheral nerve involvement. Psychotic disorder was the most common symptom, which was observed in 6 patients (6/6) in total. More than half of the patients experienced movement disorders, altered consciousness, sleep disturbances or headaches during the course. Seizures occurred in only a minority of patients.

Together with the 2 previously reported patients, 80% (8/10) of pediatric CASPR2 associated AE were older than 8 years of age at onset (12, 13). This age distribution feature was not found in MoS which is another common phenotype in children (4). This discrepancy may be due to the small total number of reported cases, and additional clinical data are still needed to summarize the epidemiological features of the disease in the pediatric population. A previous cohort study showed differences in CASPR2 antibody subtypes between patients with AE and with MoS or NMT. IgG1 antibodies were detected in 58.8% of all CSF IgG4 antibodies-positive AE patients. IgG1 antibodies were detected in the serum of all AE patients along with IgG4 antibodies in 91.7% patients. Patients with NMT or MoS had mainly IgG1 subtype in sera (83.3%) in association with IgG4 in 41.7% of the cases (15). Antibody types were not analyzed in pediatric patients. Whether antibody types can be used to explain the differences in clinical phenotypes between age groups needs to be further explored.

The rate of positive cranial MRI in CASPR2 antibody-associated disease is about 53.1% (8). In contrast, the positivity rate is about 20–30% in patients with a clinical spectrum of encephalitis (2). Imaging abnormalities include encephalitis or T2 hyperintensity in medial temporal lobes, hippocampal atrophy, medial temporal lobe sclerosis and hippocampal sclerosis. Cerebellar atrophy has also been reported in isolated cases (7). MRI scans of the pediatric population showed three cases of T2 high signal in the brain, two cases of meningeal enhancement, one case of cauda equina thickening enhancement, and one case of cerebral atrophy (4, 12–14). Interestingly, three patients with abnormal brain MRI in a pediatric case series study had negative initial imaging results (4). All patients in our cohort underwent MRI within 4 weeks of onset, and only one patient exhibited focal demyelination of the bilateral frontal lobes. No enhancement scans were performed in all patients and only one patient had a repeat cranial MRI. Therefore, we were unable to clarify the presence of meningeal enhancement or early MRI negativity.

When serum antibody titers are at low levels, there is a risk of false positives (16, 17). This was the case in the three patients excluded from this study who presented clinically with transient psychotic symptoms or cerebellar ataxia. In our cohort, 2/6 patients met the diagnostic criteria for pediatric antibody-positive autoimmune encephalitis (5). 4/6 patients did not meet the criteria for paraclinical evidence of neuroinflammation because of the absence of CSF inflammatory changes and MRI features of encephalitis (5). Although serum CASPR2 antibodies were at low titer levels, improvement in the acute phase with immunotherapy and stable neurological status after 1–9 months supported immune-mediated injury. Previous studies have shown an approximately positive rate of 30–40% for cerebrospinal fluid and 20–30% for MRI (2). Obtaining brain tissue for biopsy is very difficult in the majority of patients. We prefer that negative cerebrospinal fluid and magnetic resonance examinations in children with CASPR2-associated autoimmune encephalitis should not be used as a basis for a diagnosis of exclusion.

Autoimmune encephalitis has the potential for recurrence. The recurrence rate of anti-N-methyl-D-aspartate receptor encephalitis was ranged from 12 to 29% (18). Approximately 16–37.5% of patients with positive anti-CASPR2 antibody experienced a relapse 2 months to several years months after the first episode (7, 9, 15). In one study, half of the recurrence cases were not treated appropriately in the first episode. Treated patients had a lower relapse rate than untreated patients, which is similar to anti-N-methyl-D-aspartate receptor encephalitis (7). The recurrence rate in Asian adults with anti-CASPR2 encephalitis was 60%, especially higher among the ones treated with corticosteroids alone (19). However, this phenomenon has not been reported in pediatric patients. After a mean follow-up of 13 months all patients had no relapse. Immunotherapy resulted in favorable outcome (mRS 0–2) with control of symptoms. The reason for the recurrence of symptoms in one patient is unknown, and we cannot exclude an association with the non-administration of high-dose methylprednisolone during initial treatment.

Antibody-associated central nervous system autoimmune diseases overlapping systemic lupus erythematosus (SLE) have been reported, but the exact pathogenesis is currently unknown (20–22). One patient presented with rash and positive for multiple autoantibodies during the acute phase of the disease in this study. The patient was diagnosed with overlapping SLE with reference to the SLE diagnostic criteria (23). The symptoms disappeared after treatment with glucocorticoids and intravenous immunoglobulin. Cyclophosphamide infusion therapy was given regularly for the treatment of SLE. In addition, some patients were found to have co-morbid neoplastic diseases including thymoma, prostatic cancer, lung adenocarcinoma, melanoma, endometrial carcinoma, and pancreatic adenocarcinoma. The proportion was more pronounced in adults and in patients with Morvan syndrome and neuromyotonia (7, 8, 15, 24). No tumors were found in our group after detailed examination. To our knowledge, neoplastic diseases were almost exclusively reported in adults.

In the prodromal phase of tuberculosis meningitis, patients experience non-specific signs and symptoms, which include malaise, headache, low-grade fever, and change in personality (25). It is reasonable to suspect tuberculous meningitis in patients with tuberculosis who have psychiatric symptoms. In this study, one patient with obsolete pulmonary tuberculosis was diagnosed with autoimmune encephalitis after detailed pathogenetic examination to exclude tuberculous meningitis. This result has implications for the etiological diagnosis of central neurological symptoms in patients with active or obsolete tuberculosis.

## Limitations and Strengths

There are some limitations in this study. The relatively short follow-up period and limited number of cases may not reflect the true recurrence rate and also make the analysis of the causes of prognostic differences difficult. When antibody tests show seropositive and cerebrospinal fluid negative, or when clinical presentation does not match the identified antibody, retesting of the sample or use of confirmatory tests (e.g., brain immunohistochemistry or cultured neurons) is recommended (1). Due to the limitations of retrospective studies, we were unable to perform the above validation. The present study provides the largest amount of clinical information to date on CASPR2-associated autoimmune encephalitis in children. It is informative for the study of clinical manifestations in the pediatric phase.

## Conclusion

Our study demonstrated the clinical features of CASPR2 antibody associated autoimmune encephalitis in a cohort of pediatric patients. Unlike Morvan syndrome, this type of clinical syndrome seemed to occur more frequently in older children in the present study. Negative cerebrospinal fluid and magnetic resonance examinations are not uncommon in CASPR2-associated autoimmune encephalitis. Most patients had favorable outcomes with aggressive immunotherapy. A few patients might overlap with other autoimmune diseases such as SLE. Combined tumors in pediatric patients are extremely rare. Further studies with long-term follow-up and large samples are necessary for generalization of epidemiological features, treatment guidance and prognostic assessment.

## Data Availability Statement

The original contributions presented in the study are included in the article/supplementary material, further inquiries can be directed to the corresponding author/s.

## Ethics Statement

The studies involving human participants were reviewed and approved by Ethics Committee of the Children's Hospital of Chongqing Medical University. Written informed consent to participate in this study was provided by the participants' legal guardian/next of kin.

## Author Contributions

CT, YJ, MZ, YH, SH, XL, and LJ contributed to the analysis and interpretation of data and references. CT, YJ, and LJ participated in the conception and writing of the paper. All authors contributed to manuscript revision, read, and approved the submitted version.

## Conflict of Interest

The authors declare that the research was conducted in the absence of any commercial or financial relationships that could be construed as a potential conflict of interest.

## Publisher's Note

All claims expressed in this article are solely those of the authors and do not necessarily represent those of their affiliated organizations, or those of the publisher, the editors and the reviewers. Any product that may be evaluated in this article, or claim that may be made by its manufacturer, is not guaranteed or endorsed by the publisher.
